# Natural Germacrane Sesquiterpenes Inhibit Osteoclast Formation, Bone Resorption, RANKL-Induced NF-κB Activation, and IκBα Degradation

**DOI:** 10.3390/ijms161125972

**Published:** 2015-11-05

**Authors:** Shengnan Qin, Estabelle Ang, Libing Dai, Xiaohong Yang, Dongping Ye, Honghui Chen, Lin Zhou, Mingli Yang, Dian Teguh, Renxiang Tan, Jun Xu, Jennifer Tickner, Nathan J. Pavlos, Jiake Xu

**Affiliations:** 1 Guangzhou Institute of Traumatic Surgery, Guangzhou Red Cross Hospital, Medical College, Jinan University, Guangzhou 510220, China; qinqinsn@163.com (S.Q.); Libingdai@126.com (L.D.); dryang1192@tom.com (X.Y.); yedongping927@126.com (D.Y.); honghchen@21cn.com (H.C.); 2 School of Dentistry, University of Western Australia, Perth, WA 6009, Australia; estabelle.ang@uwa.edu.au; 3 School of Pathology and Laboratory Medicine, the University of Western Australia, Perth, WA 6009, Australia; 20463692@student.uwa.edu.au (L.Z.); 21159333@student.uwa.edu.au (M.Y.); dian.teguh@research.uwa.edu.au (D.T.); jennifer.tickner@uwa.edu.au (J.T.); 4 Institute of Functional Biomolecules, State Key Laboratory of Pharmaceutical Biotechnology, Nanjing University, Nanjing 210093, China; rxtan@nju.edu.cn; 5 Research Center for Drug Discovery (RCDD), School of Pharmaceutical Sciences, Sun Yat-Sen University, 132 East Circle at University City, Guangzhou 510006, China; junxu@biochemomes.com; 6 Centre for Orthopaedic Research, School of Surgery, the University of Western Australia, Perth, WA 6009, Australia

**Keywords:** germacrane sesquiterpenes, osteoclastogenesis, bone resorption, RANKL, NF-κB, IκBα

## Abstract

Osteolytic bone diseases are commonly presented with enhanced osteoclast formation and bone resorption. Sesquiterpene lactone natural compounds have been found to possess anti-inflammatory and immune-modulation effects. Here, we identified three germacrane sesquiterpenes using computer-based virtual screening for the structural similarity with sesquiterpene lactone, parthenolide. We showed that natural germacrane sesquiterpene compounds A, B, and C inhibit osteoclast formation and bone resorption in a dose-dependent manner, with relative potency compound A > compound C > compound B based on their equimolar concentrations. Mechanistic studies by Luciferase reporter gene assay and Western blot analysis showed that germacrane sesquiterpene compound A inhibits RANKL-induced activation of NF-κB and IκBα degradation. This study reveals that natural germacrane sesquiterpene compounds are inhibitors for osteoclast formation and bone resorption, and provides evidence that naturally-occurring compounds might be beneficial as alternative medicine for the prevention and treatment of osteolysis.

## 1. Introduction

Increased formation and activation of osteoclasts is a major pathological feature of osteolytic bone diseases, including osteoporosis, and rheumatoid arthritis [1]. The receptor activator of nuclear factor-kappa B (NF-κB) ligand (RANKL) is a key cytokine for osteoclast formation, activation, and homeostasis [2,3]. The interaction of RANKL with its receptor RANK in osteoclast precursor cells triggers a cascade of signaling pathways, including NF-κB, MAPKs, NFAT, ionic calcium, and calcium/calmodulin-dependent kinase. Among these pathways, RANKL-induced NF-κB signaling plays an essential role in osteoclastogenesis [4,5,6]. Thus, RANKL signaling is considered to be a major therapeutic target for anti-catabolic drug development. Therefore, identifying novel pharmacological interventions to perturb RANKL-mediated osteoclastogenesis and bone resorption is an important step towards the prevention and treatment of osteolysis.

Over the past decade much attention has been paid to the identification of natural products that selectively interfere with the NF-κB pathway [7]. A number of plant-derived small molecules have been identified as potential inhibitors of the NF-κB pathway. These include a wide range of skeletal classes such as lignans, sesquiterpenes, diterpenes, triterpenes, and polyphenols [7]. In particular, sesquiterpene lactones have been used as folk remedies for migraine, inflammation, arthritis, and tumors [8,9,10]. For example, parthenolide, one of the major sesquiterpene lactones found in medicinal plants has anti-inflammatory [11] and anti-osteolytic properties [6,12], and can protect from septic shock [13]. A better understanding of the mechanism(s) by which sesquiterpene analogues affect osteoclasts and RANKL signaling may provide valuable insights into the novel treatment options for osteolytic diseases.

In this study, we have systemically searched the Chinese natural chemical library via computer-based virtual screening and experimental validation using combined osteoclastogenesis assays, and NF-κB and NFAT luciferase reporter gene assays, and have identified several bioactive compounds that share the basic carbon framework to the aforementioned bioactive sesquiterpene lactones. We then tested for anti-osteoclast activity and NF-κB inhibition. We demonstrate that germacrane-based sesquiterpenes inhibit osteoclast formation and bone resorption via the suppression of RANKL-induced NF-κB activation and IκBα degradation, attesting to their potential application as anti-catabolic agents for the treatment of osteoclast-mediated osteolysis.

## 2. Results

### 2.1. Germacrane-Based Sesquiterpenes A–C Inhibit RANKL-Induced Osteoclastogenesis

**Figure 1:**
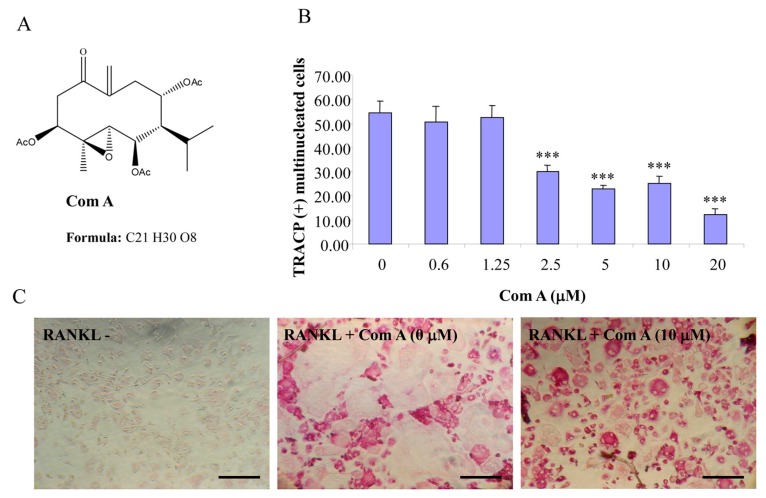
Compound A inhibits RANKL-induced osteoclastogenesis. (**A**) Chemical structure of compound A; (**B**) mouse BMMs cultured in a 96-well plate in the presence of RANKL (100 ng/mL) and M-CSF (10 ng/mL) with or without compound A for five days were fixed with 4% paraformaldehyde and stained for TRACP activity. Quantitative analysis shows the mean number of TRACP-positive multinucleated cells (MNC). (*** *p* < 0.001 compared to RANKL treated control, *n* = 3); and (**C**) representative light microscope images showing the effect of compound A on RANKL-induced osteoclast formation. (Mag = 20×, scale bar = 100 µm).

**Figure 2:**
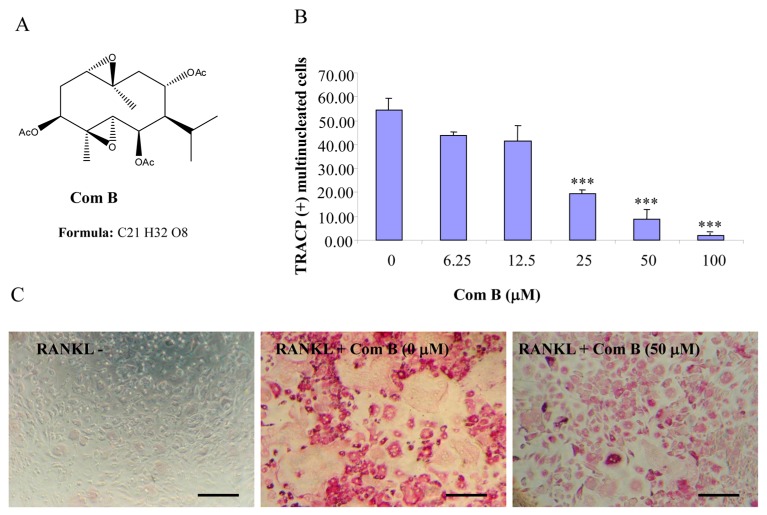
Compound B inhibits RANKL-induced osteoclastogenesis. (**A**) Chemical structure of compound B; (**B**) mouse BMMs cultured in a 96-well plate in the presence of RANKL (100 ng/mL) and M-CSF (10 ng/mL) with or without compound B for five days were fixed with 4% paraformaldehyde and stained for TRACP activity. Quantitative analysis shows the mean number of TRACP-positive multinucleated cells (MNC). (*** *p* < 0.001 compared to RANKL treated control, *n* = 3); and (**C**) representative light microscope images showing the effect of compound B on RANKL-induced osteoclast formation. (Mag = 20×, scale bar = 100 µm).

**Figure 3:**
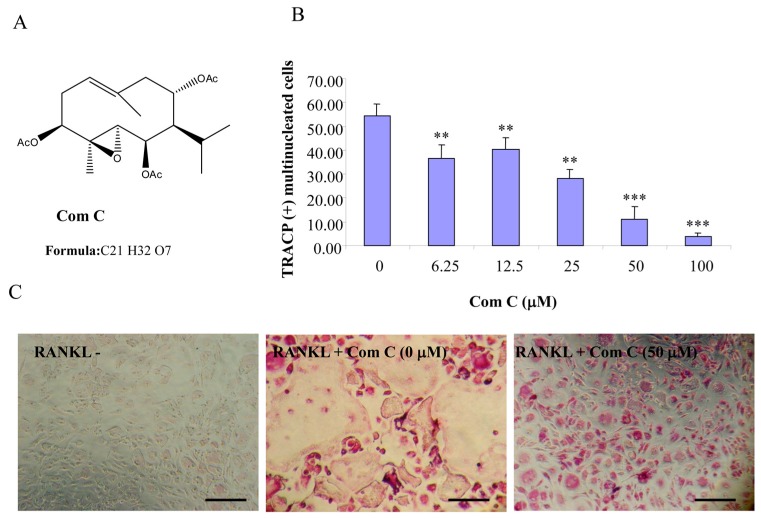
Compound C inhibits RANKL-induced osteoclastogenesis. (**A**) Chemical structure of compound C; (**B**) mouse BMMs cultured in a 96-well plate in the presence of RANKL (100 ng/mL) and M-CSF (10 ng/mL) with or without compound C for five days were fixed with 4% paraformaldehyde and stained for TRACP activity. Quantitative analysis shows the mean number of TRACP-positive multinucleated cells (MNC). (** *p* < 0.01 *** *p* < 0.001 compared to RANKL treated control, *n* = 3); and (**C**) representative light microscope images showing the effect of compound C on RANKL-induced osteoclast formation. (Mag = 20×, scale bar = 100 µm).

Compounds A–C possess germacrane sesquiterpene carbon skeletons composed typically of 15 carbons originated biosynthetically from three isoprenoid units. They can be assumed to be at least as flexible as parthenolide with a conjunction between ten- and five-membered rings (Figure 1, Figure 2 and Figure 3). Inspired by the biological function of parthenolide [6,12], compounds A–C were hypothesized to be active towards RANKL-induced osteoclastogenesis. To address such an assumption, we incubated BMM treated with RANKL in the presence of various concentrations of compounds A–C and evaluated the formation of OCL cells. TRACP positive cells with more than three nuclei were scored as OCL cells. BMM cultured in the presence of M-CSF and RANKL form multinucleated TRACP-positive OCLs. Separate addition of compounds A–C into BMM cultures showed dose-dependent inhibition of osteoclast formation as measured by the TRACP positive multinucleated cells (Figure 1, Figure 2 and Figure 3). Notably, OCL in cultured that were treated with compounds A–C were smaller than control OCL. There was no observable cell loss observed at concentrations up to 100 µM. These data thus suggest that compounds A–C inhibit RANKL-induced osteoclastogenesis, with a relative potency of compound A > compound C > compound B, based on the IC_50_ values of the inhibition of osteoclastogenesis. Thus, for the subsequent studies, compound A was chosen as a prototype to further examine its mechanism of action.

### 2.2. Compound A Inhibits Osteoclastic Bone Resorption

**Figure 4:**
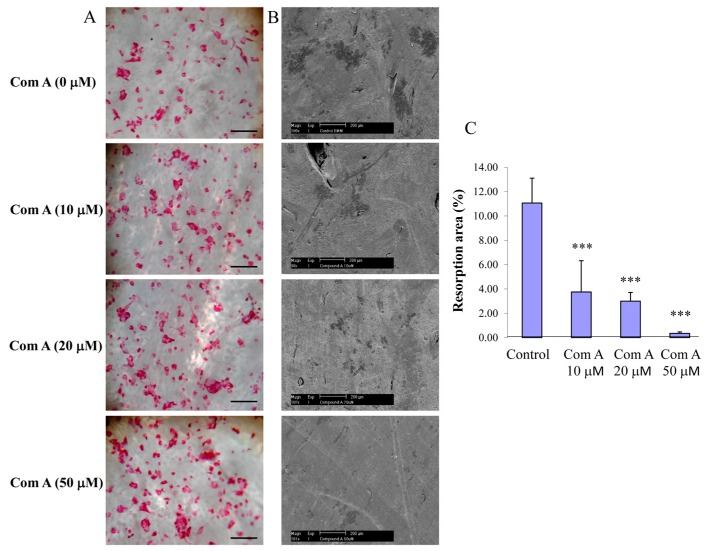
Inhibition of bone resorption by compound A. Preformed BMM derived osteoclasts were seeded on bone slices in the presence and absence of compound A for 48 h at 37 °C to initiate bone resorption. (**A**) Representative images showing TRACP positive OCL cells present in the surface of bone slice; (Mag = 20×, scale bar = 100 µm); (**B**) Representative SEM images of bone resorption (Mag = 10×, scale bar = 200 µm); and (**C**) resorption area expressed as total bone area normalized with osteoclast numbers (*** *p* < 0.001 compared to control, *n* = 3).

To test the effect of compound A on osteoclastic bone resorption, preformed BMM-derived osteoclasts were seeded onto bovine bone slices and, following attachment (~4 h post-seeding), compound A was added to the culture at varying concentrations (10–50 µM). Bone slices were retrieved after incubation for a further 48 h, and bone resorption pits were visualized and scored by scanning electron microscopy, as described in the methods. Treatments of cultures with compound A attenuated osteoclastic bone resorption (Figure 4B,C). Noteworthy, this impairment of bone resorption was not due to cell death of OCL cells as the total number of TRACP-positive cells per bone slice was not significantly different after the treatment of compound A (Figure 4A). Taken together, these experiments indicate that compound A inhibits osteoclastic bone resorption in a dose-dependent manner.

### 2.3. Compound A Suppresses RANKL-Induced Activation of NF-κB and IκB-α Degradation.

**Figure 5:**
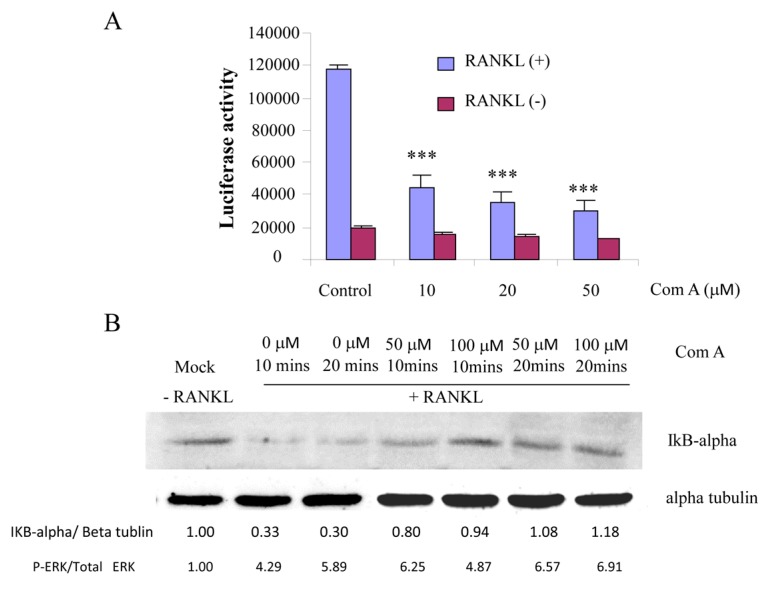
Compound A suppresses RANKL-induced NF-κB activation, and reduces IκB-α degradation. (**A**) RAW264.7 cells stably transfected with the 3kB-Luc-SV40 reporter gene were pre-treated with varying doses of compound A for 1 h followed by RANKL (100 ng/mL) stimulation. Luciferase activity in the lysates was determined after 8 h of RANKL stimulation. Each bar represents the mean ± SE from triplicate wells (*** *p* < 0.001 compared to the control, *n* = 3); (**B**) RAW264.7 cells were pre-treated with compound A for 1 h prior to RANKL (100 ng/mL) stimulation for 0, 10, and 20 min. Proteins extracted from whole cells were separated and transferred onto nitrocellulose membranes, which were then blocked and probed with antibodies to IκB-α and α-tubulin. Bands were visualized by ECL and semi-quantified by densitometry. Results shown are a representation of one of three experiments performed. The levels of IκB-α proteins are shown as a ratio to α-tubulin.

To examine the inhibitory effect of compound A on NF-κB transcriptional activity, RAW264.7 cells stably transfected with an NF-κB luciferase reporter construct [14] were stimulated with RANKL in the presence of absence of compound A. RANKL (100 ng/mL) treatment alone increased luciferase activity. Pre-treatment (1 h) of the cells with compound A at the indicated concentrations prior to RANKL stimulation (8 h) resulted in a significant dose-dependent reduction in NF-κB luciferase activity (Figure 5A).

Finally, we tested the effect of compound A on RANKL-induced IκB-α degradation. IκB-α degradation was typically detected at 10 and 20 min post-RANKL-stimulation as compared to unstimulated controls. The addition of compound A, however, resulted in a quantifiable suppression of IκB-α degradation, as seen in a time-dependent manner (10 and 20 min) (Figure 5B).

## 3. Discussion

In search for natural compounds that have anti-osteoclast and bone resorption effects, we show here that germacrane sesquiterpenes, compounds A–C, inhibit osteoclast formation, and bone resorption via the suppression of RANKL-induced activation of NF-κB and IκB-α degradation. Understanding the cellular and molecular mechanism by which these germacrane sesquiterpenes inhibit osteoclasts might prove useful for the treatment of osteoclast-mediated disorders, like osteoporosis.

NF-κB signaling has been shown to play an important role in osteoclastogenesis as NF-κB p50^−^/^−^ and p52^−^/^−^ double-knockout mice exhibit severe osteoporosis due to failure of osteoclast formation [15,16]. Activation of NF-κB has been seen in a number of osteolytic conditions with overproduction of osteoclasts [4,6]. In the present study, compounds A–C were shown to inhibit RANKL-induced NF-κB-activity and differentiation of osteoclasts. NF-κB proteins are present in the cytoplasm in interaction with inhibitory proteins known as inhibitors of NF-κB (IκB’s). Following cell stimulation by appropriate cytokines, IκB proteins become phosphorylated, ubiquitinated, and subsequently degraded by proteasome-mediated pathways [17]. The degradation of IκB allows NF-κB proteins to translocate to the nucleus and bind to their cognate DNA binding sites [17] which regulate the transcription of a large number of genes required for osteoclast differentiation and function [4,6]. Other sesquiterpene phytochemicals were also found to inhibit NF-κB by targeting IκB kinase complex, upstream molecules of IκBα [11]. In line with these findings, we have found that germacrane sesquiterpenes, compounds A–C, attenuate RANKL-induced IκBα degradation. However, whether these compounds affect other signaling molecules that can impact osteoclast formation and bone resorption remains to be defined.

Previous studies have shown that sesquiterpene lactones exert anti-inflammatory effects [8] and are able to suppress cardiovascular derangement induced by endotoxin shock. In line with these finding, parthenolide, a sesquiterpene lactone, can block lipopolysaccharide-induced osteolysis [12] and polyethylene particle-induced peri-implant osteolysis [18,19]. The inhibition of NF-κB to suppress osteoclast formation and bone resorption thus represents a promising therapeutic target for the inhibition of osteolysis.

Parthenolide is a germacranolide member of the sesquiterpene lactone family. It has a 10-membered macrocycle fused covalently with a γ-lactone ring, which was not possessed by compounds A–C. However, each of compounds A–C has three ester groups that may function biologically, as does the parthenolide lactone motif. Thus, the present investigation suggests that ester residues in the germacrane-based framework could play roles in the biological function. Further understanding of the pharmacological mechanism(s) of sesquiterpene analogues in bone biology may prove important towards the development of new and effective approaches to prevent and treat osteolytic bone diseases.

## 4. Materials and Methods

### 4.1. Natural Compounds

The following compounds were kindly provided by Renxiang Tan in Nanjing University, Nanjing, China.

### 4.2. Germacrane Sesquiterpenes Compound A (Purity > 97%)

CA Index Name: 11-Oxabicyclo[8.1.0]undecan-4-one, 2,7,9-tris(acetyloxy)-1-methyl-5-methylene-8-(1-methylethyl)-, (1*R*,2*R*,7*R*,8*S*,9*S*,10*R*)-rel-(+)-(9CI)

Other Names: 3β,6β,8α-Triacetyl-4β,5α-epoxy-1-oxogermacr-10(14)-ene isolated from Germacrane sesquiterpene esters from *salvia roborowskii* [20].

Germacrane sesquiterpenes Compound B (Purity > 97%).

CA Index Name: 5,12-Dioxatricyclo[9.1.0.04,6]dodecane-2,8,10-triol, 1,6-dimethyl-9-(1-methylethyl)-, triacetate, (1*R*,2*R*,4*R*,6*R*,8*R*,9*S*,10*S*,11*R*)-rel-(+)-(9CI).

Other Names: 3β,6β,8α-triacetoxy-4β,5α:1α,10β-diepoxygermacrane isolated from Germacrane sesquiterpene esters from *salvia roborowskii* [20].

### 4.3. Germacrane Sesquiterpenes Compound C (Purity > 97%)

CA Index Name: 11-Oxabicyclo[8.1.0]undec-6-ene-2,4,9-triol, 6,10-dimethyl-3-(1-methylethyl)-, triacetate, (1*R*,2*S*,3*S*,4*R*,6*E*,9*R*,10*R*)-rel-(-)-(9CI).

Other Names: 3β,6β,8α-Triacetyl-4β,5α-epoxygermacr-1(10)-ene isolated from Sesquiterpene esters from *salvia roborowskii* [20].

### 4.4. Media and Reagents

RAW264.7 cells were obtained from the American Type Culture Collection (Rockville, MD, USA). Alpha modified of Eagle’s Medium (α-MEM) and fetal bovine serum (FBS) was obtained from TRACE (Sydney, Australia). Rabbit anti-mouse IκB-α(c-21) polyclonal antibody and anti-mouse α-tubulin monoclonal antibody were purchased from Santa Cruz Biotechnology (Santa Cruz, CA, USA). Luciferase assay system was obtained from Promega (Sydney, Australia). Recombinant GST-rRANKL protein was expressed and purified as previously described [21].

### 4.5. Cell Culture

Primary bone marrow macrophages (BMM) were isolated from the long bone of C57BL/6 mice and grown in α-MEM supplemented with 10% heat inactivated FBS, 2 mM L-glutamine and 100 U/mL penicillin/streptomycin with the addition of macrophage-colony stimulating factor (M-CSF, 10 ng/mL). RAW264.7 cells were grown in α-MEM supplemented with 10% heat inactivated FBS, 2 mM l-glutamine and 100 U/mL penicillin/streptomycin without M-CSF. All cell cultures were maintained in 5% CO_2_ at 37 °C.

### 4.6. In Vitro Osteoclastogenesis Assay

Freshly isolated mouse BMMs were cultured with M-CSF (10 ng/mL) for the first 2–3 days. 8 × 10^3^ cells/well BMMS were seeded onto a 96 well plate in the presence of M-CSF (10 ng/mL) and GST-rRANKL (100 ng/mL). Medium was replaced every 2–3 days, cultures were fixed with 4% paraformaldehyde in phosphate buffered saline (PBS) at day five of culture. Fixed cells were and then washed four times with PBS and stained for tartrate-resistant acid phosphatase (TRACP) using the Diagnostic Acid Phosphatase kit (Sigma). TRACP positive multinucleated cells that have more than three nuclei were scored as osteoclast-like cells (OCLs).

### 4.7. Bone Resorption Pit Assay

Approximately 200 BMM-derived osteoclast like (OCL) cells were seeded onto bovine bone slices in the presence and absence of germacrane sesquiterpenes compound A. After culturing for 48 h at 37 °C, bovine bone slices were incubated for 2 h in 2 M NaOH and OCLs were removed by mechanical agitation and sonication. Resorption pits were visualized under Philips XL30 scanning electron microscope (SEM) and the percentage of bone surface area resorbed quantified using Image J software (National Institutes of Health, Bethesda, Rockville, MD, USA) [22].

### 4.8. NF-κB Luciferase Reporter Gene Assay

To examine NF-κB activation, RAW264.7 cells stably transfected with a luciferase reporter gene [14], were plated in 24-well plates at a density of 1 × 10^5^ cells/well and pre-treated with germacrane sesquiterpenes compound A for 1 h, followed by GST-rRANKL (100 ng/mL) stimulation for 8 h. Cells were harvested and luciferase activity measured using the Promega Luciferase Assay System according to the manufacturer’s instructions (Promega, Madison, WI, USA).

### 4.9. Western Blot Analysis of IκBα

Proteins were separated by SDS-PAGE gel and electroblotted onto nitrocellulose membranes (BioRad). Membranes were blocked with 5% (*w*/*v*) skim milk powder (SMP) in TBST (10 mM Tris, pH 7.5, 150 mM NaCl, 0.1% {*v*/*v*} Tween 20) and then probed with IκBα and α-tubulin (Santa Cruz Biotechnology Inc., Dallas, TX, USA) in 1% (*w*/*v*) SMP in TBST. After three washes with 1× TBST, membranes were incubated with HRP-conjugated secondary antibodies diluted 1/5000 in 1% (*w*/*v*) in TBST. The membranes were then developed and signals collected using the ECL system (Amersham Pharmacia Biotech, Sydney, Australia).

### 4.10. Statistical Analyses

Data presented were representative results from a triplicate set of three independent experiments or the mean ± SEM of those experiments. Student’s *t* test was used to test statistical significance between groups. A *p*-value of <0.05 was considered to be statistically significant.

## 5. Conclusions

Development of strategies to control the formation or activities of osteoclasts has been a major focus on the suppression of osteolysis. This study explores the effects and mechanism of a series of novel inhibitors in the suppression of osteoclastogenesis, which might serve as a potential treatment for osteoporosis. We found that natural germacrane sesquiterpenes are capable of inhibiting osteoclast formation and bone resorption, thus providing evidence that these naturally-occurring compounds might be beneficial as alternative medicine for the prevention and treatment of osteolysis.
